# Single center experience in localization of insulinoma by selective intraarterial calcium stimulation angiography - a case series of 15 years

**DOI:** 10.3389/fendo.2024.1305958

**Published:** 2024-10-21

**Authors:** Sándor Halmi, Eszter Berta, Ágnes Diószegi, Lívia Sira, Péter Fülöp, Endre V. Nagy, Ferenc Győry, Zsolt Kanyári, Judit Tóth, Harjit Pal Bhattoa, Miklós Bodor

**Affiliations:** ^1^ Division of Endocrinology, Department of Medicine, Faculty of Medicine, University of Debrecen, Debrecen, Hungary; ^2^ Doctoral School of Health Sciences, University of Debrecen, Debrecen, Hungary; ^3^ Department of Clinical Basics, Faculty of Pharmacy, University of Debrecen, Debrecen, Hungary; ^4^ Division of Metabolism, Department of Medicine, Faculty of Medicine, University of Debrecen, Debrecen, Hungary; ^5^ Department of Surgery, Faculty of Medicine, University of Debrecen, Debrecen, Hungary; ^6^ Division of Radiology and Imaging Science, Department of Medical Imaging, Faculty of Medicine, University of Debrecen, Debrecen, Hungary; ^7^ Department of Laboratory Medicine, Faculty of Medicine, University of Debrecen, Debrecen, Hungary

**Keywords:** insulinoma, selective intraarterial calcium stimulation, ASVS, nesidioblastosis, hyperinsulinemic hypoglycemia, pancreas, functioning neuroendocrine tumor

## Abstract

**Background:**

Insulinomas are rare insulin-secreting neuroendocrine neoplasms of the pancreas. First-line treatment is the surgical removal of the tumor, however, the localization with standard imaging techniques is often challenging. With the help of selective intraarterial calcium stimulation the insulinoma’s localization can be narrowed down to one third of the pancreas which the selected artery supplies.

**Objective:**

We aimed to prove the usefulness of the calcium stimulation test in case of 9 patients treated between 2006 and 2021 diagnosed with endogenous hyperinsulinemic hypoglycemia confirmed by fasting test, where conventional imaging methods, like transabdominal ultrasound, CT or MRI failed to detect the source of hyperinsulinemia.

**Methods:**

We performed selective intraarterial calcium stimulation with angiography with calcium gluconate injected to the main supporting arteries of the pancreas (splenic, superior mesenteric and gastroduodenal arteries); blood samples were obtained from the right hepatic vein before, and 30, 60 and 120 seconds after calcium administration.

**Results:**

With selective angiography we found a significant elevation of insulin levels taken from the right hepatic vein in five of the nine cases. On histopathology, the lesions were between 1-2 cm, in one case malignancy was also confirmed. In four patients we found a significant rise of insulin levels obtained from all catheterized sites, which confirmed the diagnosis of nesidioblastosis. In three cases no surgery was performed, and the symptoms relieved with medical treatment.

**Conclusions:**

Selective intraarterial calcium stimulation remains an important tool in localization of the source of insulin excess, especially in cases where other diagnostic modalities fail.

## 1 Introduction

Insulinoma is a rare neoplasm of the pancreatic beta cells with an estimated incidence of 1-4/1 million (1, 2). Despite its rare occurrence, insulinoma is the most common functioning neuroendocrine tumor of the pancreas. Most insulinomas are sporadic, however, 5-10% of the cases can also present as part of multiple endocrine neoplasia type 1 and 4, or rarely neurofibromatosis type 1 or tuberous sclerosis (3). The cases with underlying endocrine tumor syndromes need even more stringent medical attention as 16-25% of the MEN-1 syndrome cases are malignant (4, 5).

Sporadic insulinomas develop mainly in middle-aged patients, but they can occur at any age with a female predominance (60%); 87-94% of insulinoma cases are benign and solitaire (2, 3, 6).

The appropriate diagnosis of insulinoma is often delayed and is established only years after the first appearance of the clinical symptoms (7, 8). The average time until biochemically verified diagnosis is at least 2 years, but can often take 5 or more years, or even longer, and repeated hypoglycemic episodes can lead to damage of autonomic nervous system (7, 9, 10). Weight gain is also a common finding occurring in 39-50% of patients (7, 8, 11).

The biochemical diagnosis of endogenous hyperinsulinemic hypoglycemia must be obtained. The primary suspicion for insulinoma should raise when Whipple’s triad is present (7, 12). The suspected diagnosis based on the presence of Whipple’s triad needs to be verified with a method successfully detecting 99% of the cases, namely the up to 72 hours long fasting test with concurrent measurements of beta-cell polypeptides (insulin >4 µU/mL, C-peptide >0.2 nmol/L and proinsulin >5 pmol/L) at the time of hypoglycemia (3, 7).

In most cases surgical removal of the tumor is curative. However, the localization of the tumor can be quite challenging with the widely accessible conventional imaging methods as insulinomas are usually smaller than 2 centimeters in diameter (13, 14). In patients with MEN-1 syndrome insulinomas are between 1-3 cm-s and can be multifocal (14).

According to the equal distribution of beta-cells in the pancreas, insulinomas can develop anywhere within the organ, while extrapancreatic tumors are very rare (<2%) (14–16).

One goal during surgery is to reduce the exocrine and endocrine functional loss of the pancreas; parenchyma-sparing partial pancreatectomy or tumor enucleation can only be performed after proper localization of the insulinoma. Conventional non-invasive imaging techniques like transabdominal ultrasound, contrast-enhanced CT and MRI can localize the tumor properly with detection rates of 9-63%, 63-94% and 60-90%, respectively (3). In some cases, none of the above-mentioned procedures can localize the tumor and there is need for additional diagnostic procedures. Somatostatin receptor scintigraphy has a 47-60% sensitivity (3, 17). Endoscopic ultrasound sensitivity can reach 92.6%, meanwhile the most reliable method of identifying insulinomas up to date is ^68^Ga-Exendin-4 PET/CT with an accuracy of 97.7% (18, 19).

With invasive techniques, like endoscopic ultrasound or selective intraarterial calcium stimulation with venous sampling (ASVS) the detection rate increases, although the usefulness of these examinations is highly dependent on the centers’ facilities and the examiners’ experience. Pre-operative localization is essential, as 9-23% of insulinomas cannot be found by intraoperative inspection and palpation (14).

The currently used ASVS procedure is based on the observation that intravenous calcium stimulates the production and release of native insulin from the hyperfunctioning pancreatic β cells, an effect not seen in case of normal β cells (3). During the test calcium stimulation is performed through the catheterized major pancreatic arteries and blood samples are collected from the right hepatic vein (20).

The advantage of the ASVS is that besides establishing the localization of the insulinoma, it complements the morphological picture with functional information, thus the sensitivity of the procedure is reported 62.5-100% with a specificity of 89.2% (14).

In our retrospective study we examined the usefulness of ASVS in patients with hyperinsulinemic hypoglycemia, where the standard imaging methods could not find the exact localization of the tumor within the pancreas.

## 2 Patients and methods

### 2.1 Patients

Nine patients treated between 2006 and 2021 at the Division of Endocrinology, University of Debrecen were retrospectively analyzed. Each patient presented the clinical symptoms characteristic for insulinoma, and the diagnosis was supported by standard biochemical tests.

The diagnosis of the endogenous hyperinsulinemic hypoglycemia was confirmed by the detection of symptomatic hypoglycemia accompanied by documented biochemical hypoglycemia (blood glucose level below 2.5 mmol/l) and elevated insulin and C peptide levels (above 4 µU/mL and 0.2 nmol/L, respectively) during 72 hours fasting.

We included only patients in whom conventional imaging methods, like transabdominal ultrasound, CT or MRI failed to detect the source of hyperinsulinemia. Patients with proven factitious hypoglycemia caused by glucose-lowering drugs or with a history of diabetes mellitus were excluded.

### 2.2 Methods

The aim of the study was to evaluate the use of ASVS in case of nine patients previously diagnosed with insulinoma by clinical symptoms and confirmed by fasting test. With an aim to achieve information about the proper localization of the insulinoma we performed selective angiography with calcium stimulation. During selective angiography, after the punction of the right femoral artery, the gastroduodenal, superior mesenteric and splenic arteries were, one after the other, catheterized. Four ml of 10% calcium gluconate was administered to each artery. The sampling catheter was guided through the right femoral vein and placed into the right hepatic vein. Samples were obtained after selective stimulations of the arteries supplying the respective pancreatic regions before calcium administration, and 30, 60 and 120 seconds after injection. The highest insulin level of these set of samples was used for comparison. A more than 1.5 times increase of the baseline insulin level was considered significant and confirmed tumor localization within the pancreas. Insulin levels were measured from serum samples at the Department of Laboratory Medicine, University of Debrecen by chemiluminescence immunoassay (CLIA) on a Liaison XL analyzer (Diasorin Inc, Stillwater, MN, USA).

## 3 Results

In this retrospective study ASVS was performed in nine patients with endogenous hyperinsulinemic hypoglycemia. The mean patients’ age was 45 ± 25 years with a 7:2 male predominance. In case of all patients, transabdominal ultrasound, CT scan and MRI failed to localize the pancreatic neuroendocrine tumor. In case of two cases (patients 1 and 2) octreotide scintigraphy was also performed and found to be negative. Since our case series encompasses a long period in which this procedure was just getting available, endoscopic ultrasound (EUS) was not performed due to lack of equipment and diagnostic expertise in our center.

**Figure 1:**
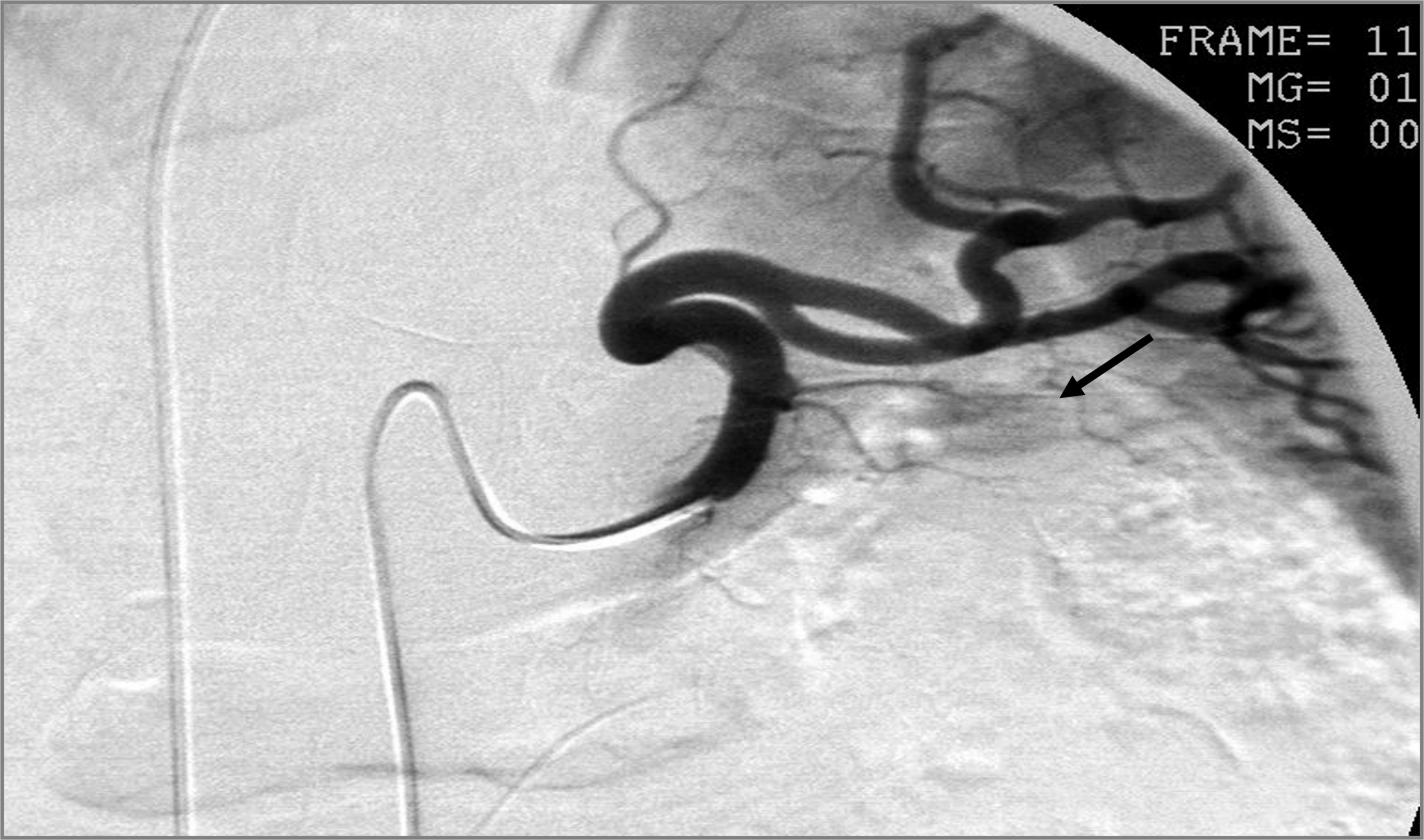
Angiography during ASVS in patient 1. After surgery, the insulinoma could be retrospectively identified on the angiography recording in the tail of the pancreas (arrow), where it was predicted by the ASVS insulin samples.

We did precisely localize the insulin overproduction source in five cases comparing the insulin content of the samples obtained from the hepatic vein (**Figure 1**). In most of our cases the patients’ serum insulin levels peaked early, as soon as 30 seconds after calcium administration with a slight decrease in the samples obtained later. These findings correlate with literature data (21).

Clinical symptoms ceased and biochemical remission was achieved after surgery in 4 patients (**Table 1**). One patient, who had several comorbidities, has been lost due to postoperative complications, the histology of the pancreatic tumor and local metastases confirmed malignant insulinoma.

**Table 1:** Results of selective intraarterial calcium stimulation and patient outcome.

Patient number									
	Patient 1	Patient 2	Patient 3	Patient 4	Patient 5	Patient 6	Patient 7	Patient 8	Patient 9
age (years)	47	29	52	70	27	66	51	34	33
gender	male	male	female	male	male	male	male	male	female
Gastroduodenal artery^1^	30.9/31.2	122.5/113.1	30.4/36.1	17.5/21.7	111.1/120.4	73.1/**>500**	204.2/**304.4**	30.5/33.2	n.a.^2^
Superior mesenteric artery^1^	16.9/13.9	24.3/**102**	33.5/**>309**	16.5/17.3	131.9/118	75/**>500**	116.9/**209.7**	43.1/**105**	17.8/**75.1**
Splenic artery^1^	17.2/**174.9**	34.8/**>291**	35.9/49.8	23.1**/>291**	119.1/**231.4**	N.A.	102.2/**>500**	42.8/**68.8**	12.1/**23.1**
Histology	insulinoma	insulinoma	insulinoma	malignant insulinoma	insulinoma	n.a.	n.a.	nesidioblastosis	n.a.
Tumor size	14-16 mm in tail region	15mm in the border of tail and body	insulinoma in the head region	insulinoma in the tail region	insulinoma in the tail region	no surgery	no surgery	n.a.	no surgery
Outcome	no hypoglycemia	no hypoglycemia	no hypoglycemia	no hypoglycemia; deceased	no hypoglycemia	medical therapy	medical therapy	medical therapy	medical therapy

^1^The values for each artery are prestimulation/poststimulation hepatic vein insulin levels (µU/mL).

^2^Unsuccessful catheterization attempt due to vasospasm.

Significantly elevated post-stimulation insulin levels are bolded.

In the other four patients the insulin levels of the right hepatic vein increased to a near similar extent after calcium stimulation of each of the three arteries. This was considered compatible with hyperplasia of the pancreatic beta-cells indicating nesidioblastosis. In case of these patients, surgery was not performed except for one case; partial pancreatectomy was followed by medical treatment. Symptoms were prevented by Ca antagonist-diazoxide combination therapy (**Table 1**).

In all our insulinoma cases the ASVS procedure was diagnostic and helped in designing and performing the surgery and also confirmed the hormonal activity of the neuroendocrine tumors.

## 4 Discussion

Insulinoma is the most common functional neuroendocrine tumor of the pancreas and is the most common cause of endogenous hyperinsulinemic hypoglycemia (7). Prevention of neurological damage may be facilitated by early localization of the autonomic focus. In most cases surgical removal is curative, although the precise localization of the tumor is often challenging, as most tumors are below 2 centimeters in diameter. On the contrary, non-functioning pancreatic neuroendocrine tumors tend to be larger, therefore are more easily detectable with conventional imaging, still being recognized later due to the lack of symptoms. Insulinomas have an outstanding surgical curability with a reported 5-year disease-free survival rate of 100% due to the relatively low percentage of malignancies (3, 22).

Nesidioblastosis was first described in children and neonates, characterized by beta-cell hyperplasia and hypertrophy. The extremely rare focal cases might be treated surgically, but in the majority of the cases pharmaceutical approach needs to be implemented (23, 24). Diffuse hyperplasia of the pancreatic islet cells is often difficult to identify with routine imaging techniques, therefore in cases of nesidioblastosis ASVS is a particularly useful diagnostic tool. During ASVS insulin level elevation was found to be significantly higher in insulinomas than in nesidioblastosis (25). We found comparably marked insulin elevation in two of the nesidioblastosis cases. EUS is a relatively new technique. It is minimally invasive, can identify neoplasms smaller than 2 cm with a high sensitivity and specificity. It also allows tissue sampling for further histological evaluation. However, the sensitivity of EUS can vary from 40% to 92.6% depending on the tumor’s location. Its accuracy is also highly dependent on the examiner’s expertise and cannot be used to assess distant metastases (18, 26, 27). Furthermore, in the absence of hyperechogenic lesions it has also limited use in the diagnosis of nesidioblastosis (23, 28, 29).

ASVS provides information about hormonal activity of the lesion as well, and thus helps localize the tumor and might help in a more precise surgical approach with a significant decrease of reoperations (30). Another study published by Morera et al. found a 90.9% sensitivity for ASVS in localizing the tumor, which is higher than the one obtained by several studies with EUS. Moreover, its sensitivity was comparable to that of intraoperative ultrasound (IOUS) performed together with palpation (31). Although it has a 47-60% sensitivity, the locally attainable octreotide scintigraphy performed in two of our cases failed to detect and localize the insulinoma (3, 17). According to several studies involving 10-20 patients, the ASVS method’s overall accuracy is around 90%. In a meta-analysis involving 339 patients a sensitivity of 93% and specificity of 86% was found (3, 32, 33). Those cases where standard imaging techniques could not detect a solitary lesion and during ASVS equally increased insulin concentrations were obtained by calcium stimulation on more than one supplying artery, were considered nesidioblastosis (24). In these patients, no tumors were found during laparotomy either. Moreover, after conservative therapy the hypoglycemic symptoms relieved, which also supports our theory.

Of course there are limitations of this technique as well. Hatoko et al. tried lower doses of calcium administration because of adverse reactions, such as nausea, hypoglycemia, hypercalcemia (34). Due to the invasive nature of the examination complication of vessel punctuation, such as bleeding or hematoma can also occur. In addition, false negative and positive results can also be found, caused by technical flaws or anatomical variants, although these are rare in case of investigations performed in skilled and well-equipped centers (35). Complications of ASVS are very rare; according to Perkov et al., complication rate is negligible, but certain precautions are needed (36). In a study of seventeen patients no complications occurred after performing ASVS, data which correlates well with our findings (37).

Novel imaging techniques using somatostatin-receptors, like octreotide-scan; ^68^Ga-DOTATATE positron emission tomography (PET) or ^68^Ga-DOTATOC PET are recently used for the detection and follow-up of neuroendocrine tumors. According to a study, Exendin-4 PET/CT was superior to ^68^Ga-DOTATATE PET/CT and ^18^F-FDG PET/CT for the localization of the insulinoma, particularly in case of small and G2 tumors (38). Exendin-4 is a molecular tracer which targets glucagon-like peptide-1 receptor (GLP-1R), which has the highest expression on insulinoma beta islet cells and consequently has a very high sensitivity and specificity in localizing pancreatic insulinomas. Sensitivity can be as high as 97.7%, which exceeds any other imaging method. ^68^Ga-NOTA-exendin-4 PET/CT is currently the most sensitive imaging method for preoperative localization of insulinomas with a sensitivity of 97.7%. However, the availability of these techniques for detecting insulinomas is limited (39, 40). The expression of GLP-1R is higher in nesidioblastosis than in normal pancreatic tissue, but lower than in insulinoma cases, which can be also a drawback of this imaging method (41).

Molecular imaging is an emerging and promising tool in the detection of insulinomas; however, a significant percentage of insulinomas do not express somatostatin receptors (41). GLP-1R is overexpressed in 93% of the cases, consequently GLP-1 PET/CT improves insulinoma detectability vastly; however, overexpression is only present in 36% of patients with metastases and/or malignant lesions, making the method less informative in the rare but more malevolent malignant cases (41–43). Moreover, metastatic insulinomas often lack GLP-1 receptors, and often SST2 receptor overexpression can be found (positive SRS scan in 73%) (44).

Albeit in almost every case the combination of different imaging techniques is required, in the preoperative phase the precise localization of the insulinomas is unachievable in 10-27% of the cases (14). In a systematic review of 6222 cases evaluated between 1960 and 2011, ASVS localized correctly 84.7% of insulinomas, when applied, with a mean sensitivity of 89.2% (14). ASVS might provide additional functional information in MEN-1 cases when multiple neuroendocrine neoplasms are present in the pancreas and distinction is needed between potential functioning and non-functioning tumors (3).

There are different viewpoints about the best methods to localize the tumor; the available diagnostic procedures are different in every center, and the success rate can be highly dependent on the centers’ preparedness and experience (3, 22, 45). In the presented case series, our institution has served as referral center for neuroendocrine tumors, which explains the high success rate in localizing insulinomas and recognizing nesidioblastosis cases, which underscores the importance of the centralized management of rare endocrine tumors like insulinoma.

To avoid the late exocrine and endocrine pancreas function insufficiency and to facilitate postoperative healing, the best curing procedure is the pancreas saving surgical intervention, for which the exact localization of the tumor is indispensable. In the most recent European Neuroendocrine Tumor Society (ENETS) guidance paper, as first-line treatment modality for patients with preoperatively localized insulinomas a minimally invasive surgical approach is strongly suggested; laparoscopic procedures are reported to be safe and effective treatment options (7, 13, 14, 46).

The prevalence of nesidioblastosis is growing; the incidence increases after bariatric surgery (23, 47). In our institution we found a relatively high number of nesidioblastosis cases among the investigated hyperinsulinemic patients. In nesidioblastosis surgical intervention does not lead to complete healing (48). Preoperative screening is fundamental to avoid unnecessary surgery. Diazoxide reduces insulin secretion by indirect action on beta-cells and enhances glycolysis. Long-acting somatostatin analogues (SSAs) (octreotide, lanreotide and pasireotide) may prevent hypoglycemia when the insulinoma tumor cells express somatostatin receptors subtype 2 (14, 17). For metastatic insulinoma cases peptide receptor radionuclide therapy (PRRT) with ^177^Lu-DOTATATE and everolimus can be considered in advanced, progressive insulinoma cases when hypoglycemia is refractory to SSAs (7).

In a recent study of Andreassen et al., the tumors of 80 patients showed less staining for insulin and proinsulin in malignant insulinoma cases vs. benign lesions, possibly due to a diminished insulin storage capacity; to the contrary, glucagon staining was present only in malignant tumors. Malignancy is also associated with a lack of staining for CgA and higher Ki-67 staining as a result of poor differentiation (15). These findings also underscore the usefulness and importance of the ASVS technique.

In our series of patients ASVS provided important functional information and could successfully localize the origin of the elevated insulin levels, which were usually higher in localized insulinomas, then in nesidioblastosis. No complications occurred during ASVS. Although the number of patients studied is relatively small, according to our results ASVS is still an effective tool when the source of insulin over-secretion cannot be localized with non-invasive imaging. This is in line with the most recent ENETS recommendation (7).

One limitation of our case series investigation is that several, recently available imaging methods were not, or just partially performed (EUS, IOUS, radionucleotide-labeled techniques), so true comparison of these methods cannot be estimated. The limited number of cases is also a handicap of our study.

## 5 Conclusions

The localization of insulinoma is fundamental, as the treatment of choice is the surgical removal of the tumor. ASVS remains a reliable tool in localization and possesses important additional functional information that are not achievable with the use of other, novel and more expensive imaging techniques.

## Data Availability

The original contributions presented in the study are included in the article/supplementary material. Further inquiries can be directed to the corresponding author/s.
